# Microgravity Level Measurement of the Beijing Drop Tower Using a Sensitive Accelerometer

**DOI:** 10.1038/srep31632

**Published:** 2016-08-17

**Authors:** T. Y. Liu, Q. P. Wu, B. Q. Sun, F. T. Han

**Affiliations:** 1 Department of Precision Instrument, Tsinghua University, Beijing, 100084, China

## Abstract

Drop tower is the most common ground-based facility to provide microgravity environment and widely used in many science experiments. A differential space accelerometer has been proposed to test the spin-gravity interaction between rotating extended bodies onboard a drag-free satellite. In order to assist design and test of this inertial sensor in a series of ground- based pre-flight experiments, it is very important to know accurately the residual acceleration of drop towers. In this report, a sensitive instrument for this purpose was built with a high-performance servo quartz accelerometer, and the dedicated interface electronics design providing small full-scale range and high sensitivity, up to 136.8 V/*g*_0_. The residual acceleration at the Beijing drop tower was measured using two different drop capsules. The experimental result shows that the microgravity level of the free-falling double capsule is better than 2 × 10^−4^*g*_0_ (Earth’s gravity). The measured data in this report provides critical microgravity information for design of the following ground experiments.

Microgravity experiments have gained more and more attention for various research fields, such as space technology, fluid mechanics, combustion science, biophysics, materials and fundamental physics^1^,^2^,^3^,^4^,^5^. We have proposed a scheme for testing spin-gravity interaction between rotating extended bodies in a drag-free satellite, which needs a differential electrostatic accelerometer as the core space experiment instrument^6^. This ultra-low range and sensitive space accelerometer will undergo a series of ground-based pre-flight experiments to verify its function and performance. Currently, various facilities are available to perform microgravity experiments near the ground, such as drop towers or drop wells, parabolic flights, balloon-drops and sounding rockets^7^,^8^,^9^. The small residual gravity, low cost and high throughput of experiments^10^ make the drop tower become an ideal choice for our ground-based test. Although the equivalence principle could be tested directly in a drop tower^11^, we aim to test the new equivalence principle in space to reach a better accuracy and the Beijing drop tower will be utilized to perform various ground-based microgravity experiments. In this case, before loading the costly and precise differential electrostatic accelerometer in the drop tower, the basic performance of the drop tower needs to be measured separately in order to determine whether the microgravity environment provided by the drop tower meets the ground-test requirements of the differential electrostatic accelerometer. Therefore, it is necessary to accurately measure the residual acceleration experimentally rather than a theoretical estimation or indirect predication, which is the goal in this report.

The basic performance of the Beijing drop tower, which is located at the National Microgravity Laboratory, Chinese Academy of Science (NMLC), is presented in this report. The drop capsule starts falling at 83 m above the ground, and the deceleration unit works from about 22 m above the ground to 0 m, as Fig. 1. Two types of drop capsules can be selected by users for different microgravity levels. One type is a single capsule where it falls directly in the air. The other is a double capsule comprised of an inner small capsule and an outer big capsule. The outer capsule falls directly in the air while the inner capsule falls inside the low air-pressure outer capsule^12^. The effect of air drag on drop tower experiments can be attenuated greatly by evacuating air or using double capsules, which helps to reduce residual acceleration and achieve better microgravity level^13^. The two types of drop capsules provide different microgravity levels. As the Beijing drop tower is currently used for many microgravity experiments, the results provided in this report will also be helpful to other researchers in analysing their experimental data.

Several other drop tower facilities have announced their theoretical residual acceleration. Zero Gravity Research Facility at NASA Glenn Research Center uses a vacuum chamber to reach a residual acceleration below 10^−5^ *g*_0_^14^. JAMIC drop tower in Japan, the biggest one in the world, uses double capsule to reach 10^−6^ *g*_0_^15^, although it is not in operation currently. The drop tower at Queensland University of Technology reaches 10^−4^ *g*_0_^14^ and the one at Portland State University is 2 × 10^−4^ *g*_0_^16^. Among them, the Bremen drop tower at ZARM can provide the best microgravity condition, by using a double capsule system, down to 10^−6^ *g*_0_^10^. However, most of these results are theoretical predication rather than experimental data.

There are mainly two methods to measure the residual acceleration level for drop towers^10^. One is utilizing a high-resolution accelerometer mounted on the drop capsule to measure the residual acceleration directly. The other is to record the displacement-time data of the free-falling capsule using a laser interferometer, and then the residual acceleration can be calculated by comparing the measured and nominal acceleration of gravity. The second method needs a mirror fixed outside the inner drop capsule, a glass window on the outer capsule, and a laser interferometer mounted in the tower. As it is costly and time-consuming to update the associated drop tower facility, the first test method will be a better choice.

In this report, a sensitive instrument for microgravity measurement is presented based on a high-performance servo quartz accelerometer. The dedicated interface electronics design provides extremely high sensitivity by conditioning its full-scale range. The calibration of the instrument was performed on a precision turntable, which indicates a scale factor up to 136.84 V/*g*_0_ and an overall accuracy better than 4 × 10^−5^ *g*_0_. Two kinds of accelerometer-based experiments, using the single capsule and the double capsule, were conducted to measure the residual acceleration of the Beijing drop tower. The experimental result shows that the microgravity level of the free-falling double capsule is better than 2 × 10^−4^ *g*_0_.

## Results

### Single capsule

**Figure 2:**
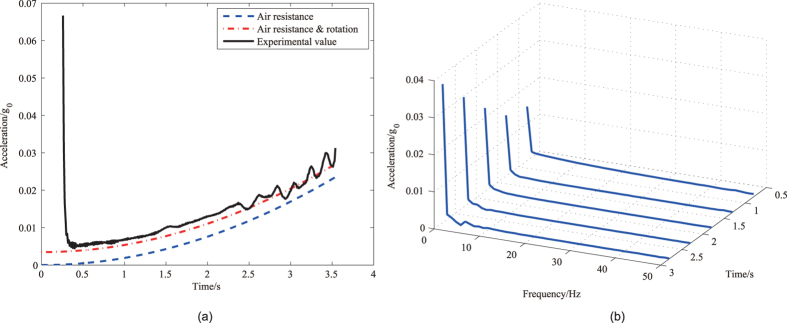
The result of the single drop capsule. (**a**) The measured acceleration vs. time in the single capsule experiment. The residual accelerations caused by air drag only and by both air drag and capsule rotation are plotted for comparison. (**b**) The waterfall diagram of the residual acceleration’s frequency for the single capsule.

A typical residual acceleration of the single drop capsule is shown in Fig. 2(a) where the duration of the free fall is nearly 3.5 s. At the beginning of the fall, the measured residual acceleration does not suddenly reduce to the theoretical predication. Then the residual acceleration increases as the air drag force is rising over time. At the end, the residual acceleration of the single capsule is less than 0.03 *g*_0_. Besides, the residual acceleration has a small range of fluctuations in the latter part. It is also worth noting that the experimental microgravity has an overall bias by comparing with the calculated residual acceleration using (3).

Figure 2(b) shows the corresponding frequency spectra of the measured acceleration data. The signal frequency components are mostly less than 5 Hz, while the bandwidth of the microgravity instrument is 24 Hz which is higher than the highest signal frequency. The measured result shows the residual acceleration of the single capsule is mainly in low frequency range (0~5 Hz) while the high frequency components are negligible.

### Double capsule

**Figure 3:**
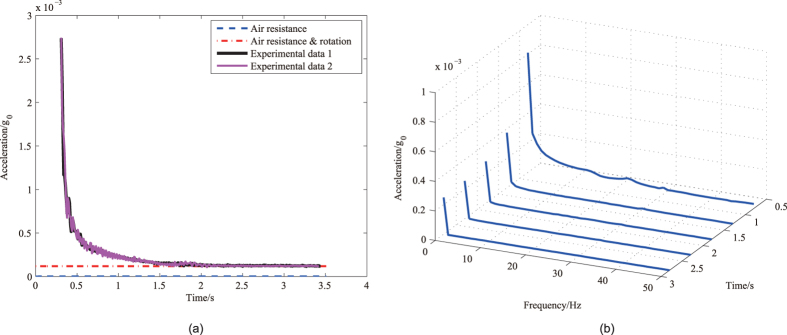
The result of the double drop capsules. (**a**) The measured acceleration vs. time in the double capsule experiment. The calculated residual accelerations caused by the air drag and rotation are plotted for comparison. (**b**) The waterfall diagram of the residual acceleration’s frequency for the double capsule.

Figure 3(a) shows the residual acceleration as a function of time during two double capsule experiments. The results in two experiments are mostly identical, which shows the experiments have good repeatability. The Fig. 3(a) shows that the maximum residual acceleration in the inner capsule is less than 2 × 10^−4^ *g*_0_. The results from both the single capsule and double capsule experiments have some similar characteristics. The measured residual acceleration in Fig. 3(a) does not suddenly reduce to its theoretical predication at the beginning, either. Besides, the measured acceleration still has an overall bias by compared with the calculated result by considering the air drag effect. However, during the double capsule experiments, the residual acceleration does not increase over time since the air drag is much weaker than the single drop capsule.

Figure 3(b) shows the corresponding frequency spectra of the measured residual acceleration. In this case, the signal frequency components are mostly less than 3 Hz, which is similar to the single capsule experiments.

## Discussion

The air drag plays an important effect on the residual acceleration. In order to facilitate the analysis of experimental results, the theoretical acceleration caused by air drag is estimated first. For the single capsule experiment, the air drag is mainly caused by the form drag. One way to express the drag force is by means of the drag equation:


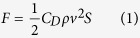


where *F* is the drag force, *C*_*D*_ is the drag coefficient, *ρ* is the air density, *v* is the falling speed, and *S* is the cross sectional area of the drop capsule. Then, the residual acceleration caused by air drag will influence the falling speed as:


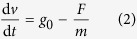


where *m* is the mass of the drop capsule and *t* is the time. Assuming the initial velocity of the drop is zero, substituting (1) into (2) yields the residual acceleration as:


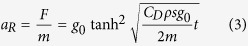


According to the capsule parameters, the maximum theoretical residual acceleration in the falling single capsule is 0.0236 *g*_0_.

The theoretical acceleration caused by air drag in the double capsule experiment can be estimated by analogous method. For the inner capsule of the double drop capsule, both the form drag and the skin friction contribute to air drag as:


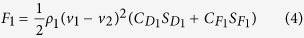


where *F*_1_ is the air drag of the inner capsule, *v*_1_ and *v*_2_ are the falling speed of the inner and outer capsules, *ρ*_1_ is the air density between the inner capsule and the outer capsule, 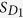
is the cross sectional area of the inner capsule, 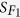 is the surface area of the inner capsule, 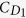 and 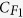 are the drag coefficient and the friction coefficient, respectively.

According to Blasius Friction Law,


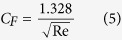


where Re is the Reynolds number. The calculation of the air drag *F*_2_ of the outer capsule is similar to the single capsule. The relationship between the speed and air drag is:


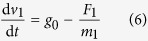



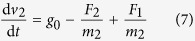


where *m*_1_ is the mass of the inner capsule and the *m*_2_ is the mass of the outer capsule. The numerical result shows that the residual acceleration caused by air drag rises from zero to less than 10^−7^ *g*_0_ during the falling process of the double drop capsule.

It should be noted that the experimental microgravity has an overall bias by comparing with the calculated residual acceleration in both the single capsule experiment and the double capsule experiment. Some other microgravity experiments showed that the falling-capsule’s rotation contributes to extra residual acceleration^10^,^17^,^18^, which is applicable to this drop tower measurement. As the accelerometer is not installed ideally at the centre of the drop capsule, any small rotation of a capsule will introduce additional acceleration which can be decomposed into two components, i.e., normal acceleration and tangential acceleration.


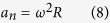



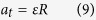



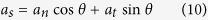


where *ω* is the angular velocity, *ε* is the angular acceleration, *R* is the distance from the accelerometer to the rotation axis, *a*_*n*_ is the normal acceleration, *a*_*t*_ is the tangential acceleration, *a*_*s*_ is the acceleration on the sensitive axis of the accelerometer and *θ* is the angle between the sensitive axis and the rotation radius. Although the experiment does not measure the angular velocity and angular acceleration of the drop capsule, we can estimate the magnitude of the rotation according to the measured bias acceleration. For instance, when *ω* = 0.07 rad/s, *ε* = 0.05 rad/s^2^, *R*_*r*_ = 1.4 m, and *θ* = 0.5 rad, then *a*_*s*_ = 4.04 × 10^−3^ *g*_0_, which has the same magnitude as the result of the single drop capsule. For the double drop capsule, as the air drag is much smaller in vacuum, the bias is partly resulted from the attainable accuracy of the instrument, and partly due to the inner capsule’s rotation. For instance, considering the dimension of the capsule, when *ω* = 0.02 rad/s, *ε* = 0.01 rad/s^2^, *R*_*r*_ = 0.4 m, and *θ* = 0.3 rad, then *a*_*s*_ = 1.36 × 10^−4^ *g*_0_.

As the experiments results show, the measured residual acceleration does not suddenly reduce to the theoretical predication at the beginning. This is mainly attributed to recovery time of the accelerometer output from 1 *g*_0_ saturation condition to its full scale range, and it’s partly due to the limited bandwidth of the interface electronics. The residual acceleration of the single drop capsule has a small range of fluctuations, which is mainly because of that the drop tower is not a smooth cylinder. The drop tower has some raised floors, so the drop tower spaces of different diameters cause falling-height dependent wind disturbance to air drag. The uneven airflow generates a disturbance on both translation and rotation motion of the falling capsule. For the double capsule experiment, as the air drag is weak on the inner capsule, the result doesn’t show obvious fluctuations as the single capsule.

The experimental results show that the microgravity level of the Beijing drop tower is better than 3 × 10^−2^ *g*_0_ and 2 × 10^−4^ *g*_0_ for the single and the double capsules, respectively. It is clear that the microgravity level of Beijing drop tower is not as good as some other drop towers such as the Bremen drop tower at ZARM which can reach 10^−6^ *g*_0_^10^. As the drop tower is used to test the space experiment instruments instead of testing the equivalence principle, the double capsule in the Beijing drop tower can provide good microgravity condition. Considering that the full-scale input range of the differential electrostatic accelerometer for the space mission can be changed by adjusting the bias voltage^19^, we can use the microgravity environment provided by the Beijing drop tower to test and verify the instrument’s function and performance by matching its range with the measured microgravity level. Hence, the results in this report provide the critical information for the instrument design and future ground experiments. For fully test of the differential accelerometer with micro-g condition, some innovative techniques, such as free flyer for the drop capsule, can be used to update the Beijing drop tower so as to achieve higher microgravity condition.

## Methods

### Instrument for microgravity measurement

#### Design

In order to measure the motion of the capsule fully, a three-axis accelerometer is needed. However, the purpose of our experiment is not to fully measure the microgravity condition of the drop tower, but to verify whether the drop tower meets the ground-test requirements of our space experiment instrument. Due to the large air resistance caused by the high vertical speed of the drop capsule, the residual component in the vertical direction is usually higher than them in two horizontal directions. So we choose a single-axis accelerometer to test the microgravity level of the Beijing drop tower along the vertical direction.

The residual accelerations for the free-falling single capsule and double capsule are estimated on the order of 10^−3^ *g*_0_ and 10^−5^ *g*_0_^13^, respectively. Thus, the instrument for microgravity measurement should have a full-scale range on the order of 10^−2^ *g*_0_ and an acceleration resolution better than 10^−6^ *g*_0_. Moreover, as a maximum shock force of about 12 *g*_0_ exists when the drop capsule is crashing into the deceleration unit^13^, the instrument should be able to resist this impact.

**Figure 4:**
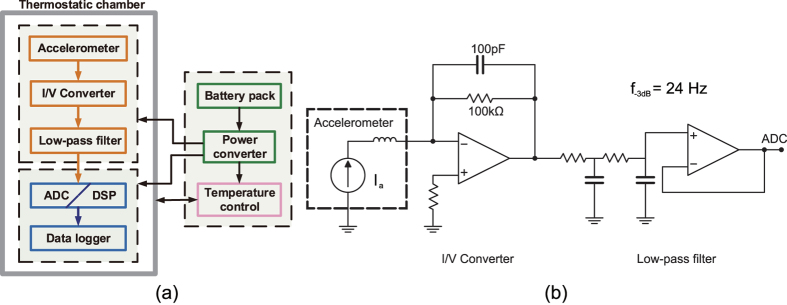
Instrument design for microgravity measurement. (**a**) Block diagram of the instrument for microgravity measurement. (**b**) The circuit diagram of I/V and low-pass filter.

The block diagram of the microgravity instrument is shown in Fig. 4(a). The core part of the instrument is a commercial quartz flex accelerometer from *Shaanxi Space-flight & the Great Wall M&C Co., LTD* and whose model is *JHT-I-A*. The scale factor of the accelerometer is about 1.3 mA/*g*_0_, whose exact value can be determined in the following calibration. It offers a satisfactory anti-impact overload capability up to 120 *g*_0_. An active current-to-voltage converter (I/V) with a relatively large sampling resistor converts the output current to a voltage signal, which results in a much higher instrument sensitivity and low full-scale range. A second-order analog low-pass filter with a cut-off frequency of 24 Hz is followed to attenuate high-frequency noise and thus achieve high acceleration resolution. A schematic of the I/V and the filter is shown in Fig. 4(b). An 18-bit analog-digital converter (ADC) converts the analog signal to a digital signal at a sampling frequency of 100 Hz. The data from ADC is processed by a digital signal processor (DSP), then sent to a data logger and recoded in a micro-SD card. Considering that ambient temperature plays a significant effect on accuracy of the accelerometer and its interface electronics, a constant temperature chamber is used to enclose the instrument core fully and ensure a stable operating temperature within 35.0 ± 0.2 °C. The necessary onboard power supplies are provided by a DC/AC inverter for heater and a linear DC/DC converter for analog and digital electronics.

#### Calibration and testing

**Figure 5:**
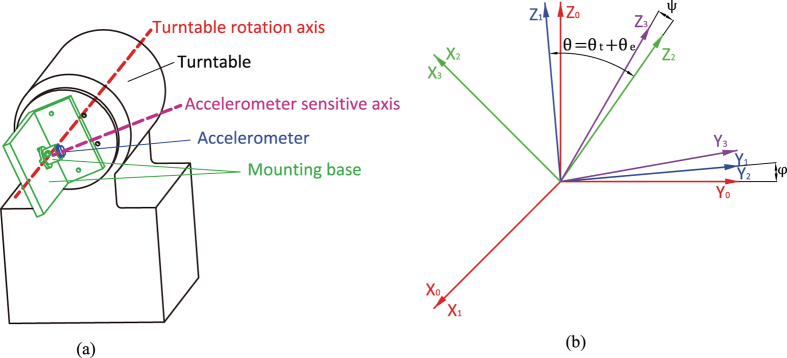
Calibration. (**a**) Calibration on a turntable (thermostatic chamber, etc. omitted). (**b**) The coordinates used in calibration.

The calibration of the instrument was performed on a precision single-axis turntable with a resolution of 0.0001°, as illustrated in Fig. 5(a). The accelerometer bias and other misalignment from the installation or the turntable should be separated. Ideally, the turntable rotation axis is in the horizontal direction Y_0_, and the accelerometer sensitive axis is in the vertical direction Z_0_ as in Fig. 5(b) if the turntable angle is 0°. In fact, the turntable rotation axis is not ideally in the horizontal planes. The coordinate system OX_0_Y_0_Z_0_ becomes OX_1_Y_1_Z_1_ when rotating a angle of *φ* around the axis X_0_, and the rotation axis of the turntable is along Y_1_. If the turntable angle is *θ*_*t*_, the sensitive axis of the accelerometer is along Z_3_ because of the misalignment angles *θ*_*e*_ and *ψ*. The coordinate system OX_1_Y_1_Z_1_ becomes OX_2_Y_2_Z_2_ when rotating a angle of *θ* including *θ*_t_ and *θ*_*e*_ around −Y_1_, and OX_2_Y_2_Z_2_ becomes OX_3_Y_3_Z_3_ when rotating a angle of *ψ* around X_2_. The acceleration components in OX_3_Y_3_Z_3_ are as:


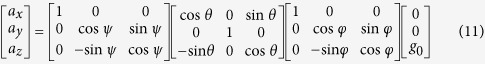


So the acceleration component in the sensitive axis of the accelerometer is


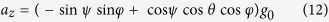


In each calibration, as *ψ*, *θ*_*e*_ and *φ* are extremely small constants, so the acceleration can be approximately expressed as


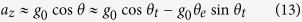


The instrument output is


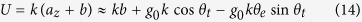


where *k* is the scale factor and *b* is the bias. In the calibration, the instrument output *U*_*i*_ is a function of the angle *θ*_*ti*_ by controlling rotation of the turntable.


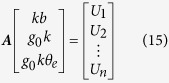



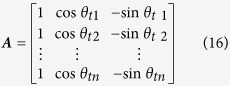


The scale factor *k* and the bias *b* can be solved using the method of least squares as


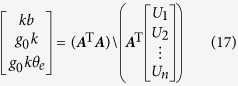


The distance between the location of calibration experiments and the location of the drop tower experiments is shorter than 2 km, which is considered to have same gravity acceleration *g*_0_, about 9.8015 m/s^2^. Totally eighteen turning angles *θ*_*ti*_ were selected, including 86°, 87°,…, 94°, and 266°, 267°,…, 274°. The turntable was held stationary for one minute at each angle, and the average value of the instrument output was recorded. The measured scale factor is 136.84 V/*g*_0_. The full-scale range is ±0.073 *g*_0_, and the resolution is 5.6 × 10^−7^ *g*_0_, which are limited mainly by the ADC.

**Figure 6:**
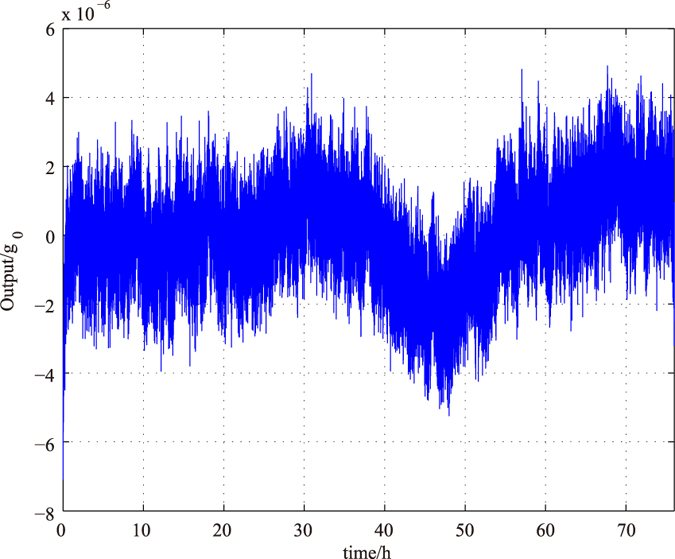
Bias stability in 75 hours.

The bias stability and repeatability of the instrument is crucial to test extremely small drop-tower acceleration. According to a 75-hour output data shown in Fig. 6, the bias drift is less than 4 × 10^−6^ *g*_0_ (max) or 1.5 × 10^−6^ *g*_0_ (1*σ*). The repeatability of the bias is better than 1.3 × 10^−5^ *g*_0_, and the repeatability of the scale factor is better than 0.03 V/*g*_0_ according to several calibration experiments, which contributes an error of less than 1.6 × 10^−5^ *g*_0_. After careful calibrations were performed, the overall accuracy of the instrument is better than 4 × 10^−5^ *g*_0_ by considering all these error sources.

### Drop tower experiment

#### Single capsule

**Figure 7:**
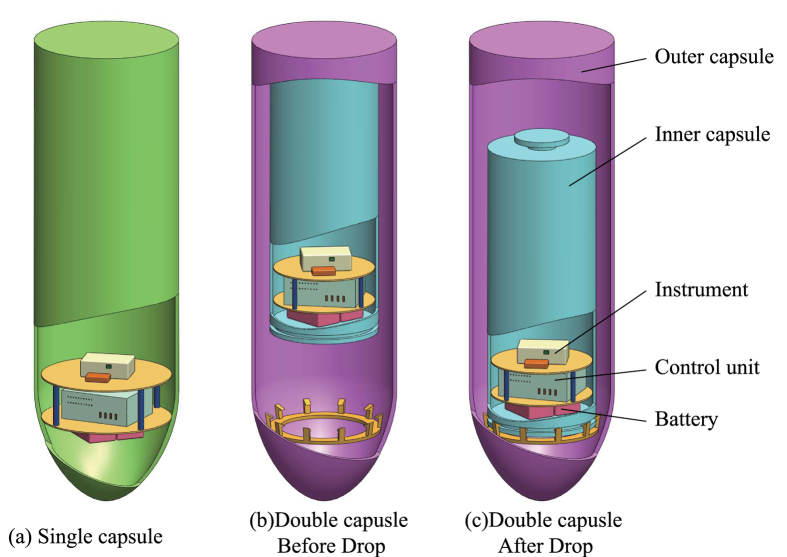
The instruments are mounted (**a**) in single capsule, (**b**) in inner capsule fixed on the top of the outer capsule before drop and (**c**) in inner capsule falling to the bottom of outer capsule after drop.

**Figure 8:**
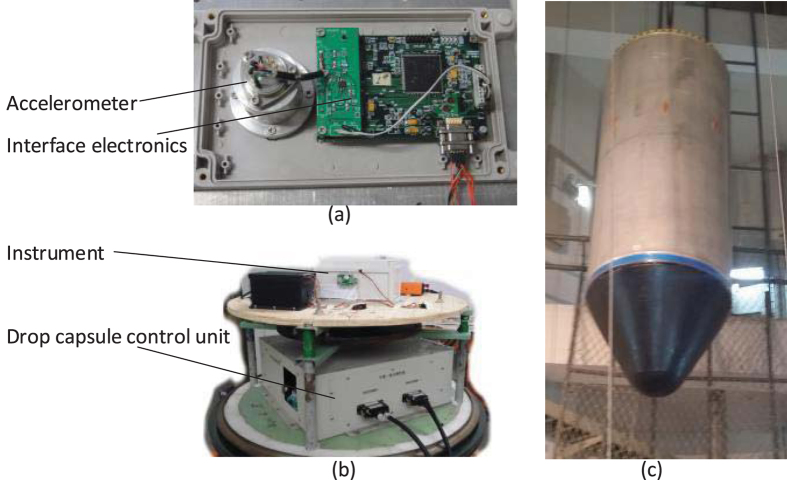
(**a**) The accelerometer and interface electronics inside a thermostatic chamber. (**b**) The instrument mounted inside the drop capsule. (**c**) Outside the drop capsule.

The instrument can be mounted in two types of drop capsule as depicted in Fig. 7 where the accelerometer sensitive axis is along the direction of the drop. When conducting experiments with the single capsule, the instrument is fixed near the radial centre of the drop capsule, as the instrument set-up shown in Fig. 8. After the instrument is powered on and passes the self-test, the capsule assembly is sealed. In this case, several small masses need to be fixed on the capsule to calibrate and balance its centre of mass. Then the drop capsule assembly is lifted up to a predetermined height and staying there for the release order. Before the drop capsule is released, a set of electromagnets will suck and hold the capsule instead of mechanical fixtures. Once the electromagnet is powered off, the drop capsule will be released and begin free falling. The time duration from sealing to releasing is usually over one hour, during which the constant temperature chamber reaches steady state and maintains at its target temperature. The single capsule falls directly at atmospheric pressure. The drop tower has been equipped a set of deceleration devices on the bottom, which prevent the capsule from huge impact. Finally, the whole experimental data can be obtained from recorded data on the micro-SD card when the capsule assembly is opened.

#### Double capsule

When performing free fall experiment with the double capsule, the experimental process is more complex as the two concentric capsules need to be operated in a vacuum. Firstly, the inner small capsule is fixed on the top of the outer big capsule after the inner capsule is sealed, and then the outer capsule is sealed. The air between the inner and the outer capsules is pumped out by a vacuum pumping station until the air pressure is less than 30 Pa. It should be noted that the final pressure could increase to about 110 Pa at the drop moment as there is a small amount of air leakage inside the outer capsule during balancing the centre of mass and lifting up to the released position. When the electromagnet is powered off, both the outer capsule and the inner capsule begin falling. At this moment, the outer capsule falls in the atmosphere while the inner one falls in the low pressure chamber between the inner and the outer capsules. As the inner drops a little faster than the outer, the inner capsule will touch the bottom of the outer capsule before the outer capsule entering deceleration region. After the inner capsule is fixed to the bottom of the outer capsule by dedicated mechanical device, both the inner and the outer fall into the deceleration devices together.

## Additional Information

**How to cite this article**: Liu, T. Y. *et al.* Microgravity Level Measurement of the Beijing Drop Tower Using a Sensitive Accelerometer. *Sci. Rep.* **6**, 31632; doi: 10.1038/srep31632 (2016).

## Figures and Tables

**Figure 1:**
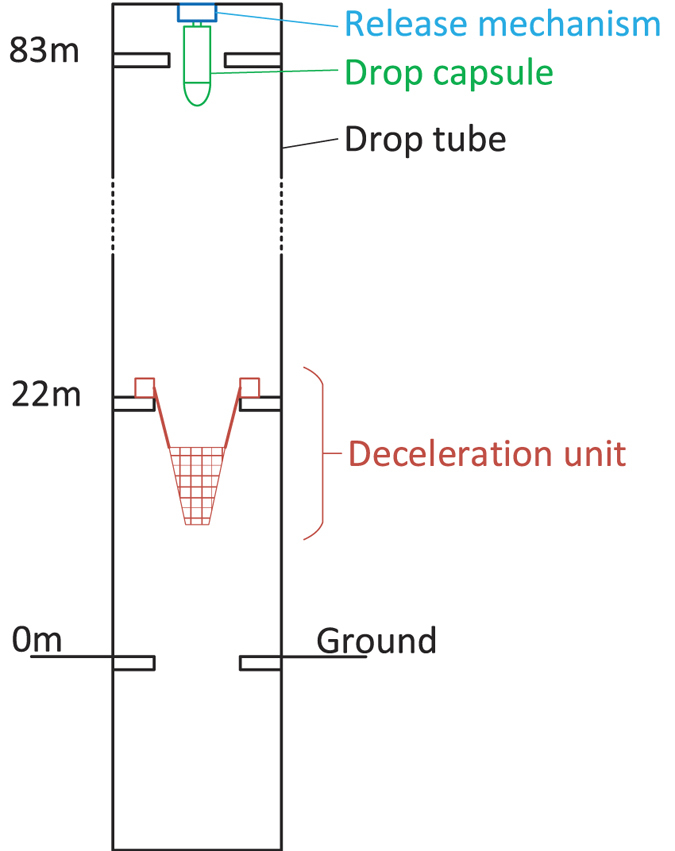
Sketch of the Beijing Drop Tower.
